# Cell Senescence in Lupus

**DOI:** 10.1007/s11926-019-0800-6

**Published:** 2019-01-14

**Authors:** Lin Gao, Maria Slack, Andrew McDavid, Jennifer Anolik, R. John Looney

**Affiliations:** 1 Allergy Immunology Rheumatology Division, Department of Medicine, University of Rochester School of Medicine and Dentistry, Rochester, NY USA; 2 Department of Biostatistics and Computational Biology, University of Rochester School of Medicine and Dentistry, Rochester, NY USA

**Keywords:** Systemic lupus erythematosus, Cellular senescence, DNA damage, Telomeres, Mesenchymal stem cells

## Abstract

**Purpose of Review:**

The concept of cellular senescence has been evolving. Although originally proposed based on studies of serum-driven replication of cell lines in vitro, it is now clear that cellular senescence occurs in vivo. It has also become clear that cellular senescence can be triggered by a number of stimuli such as radiation, chemotherapy, activation of oncogenes, metabolic derangements, and chronic inflammation.

**Recent Findings:**

As we learn more about the mechanisms of cellular aging, it has become important to ask whether accelerated cellular senescence occurs in lupus and other systemic rheumatologic diseases.

**Summary:**

Accelerated cellular aging may be one explanation for some of the excess morbidity and mortality seen in lupus patients. If so, drugs targeting cellular senescence may provide new options for preventing long-term complications such as organ failure in systemic lupus erythematosus patients.

## What Is Cellular Senescence?

In a population, aging is associated with an exponential increase in the mortality rate [1]. At the organismal levels, aging is seen as a progressive loss of function and resilience. At the cellular level, aging is associated with a number of “hallmarks” [2]. Cellular senescence, defined as stable arrest of cell cycle in association with several stereotypic phenotypic changes, is one of the hallmarks of aging. Cellular senescence was first described in 1961 as the grown arrest after 40 to 60 population doublings of human embryonic fibroblasts grown in tissue culture. With growth arrest, cells did not die but flattened out and became larger. Other characteristics of cellular senescence include: a persistent DNA damage response, activation of cyclin-dependent kinase inhibitors, enhanced secretion of pro-inflammatory and tissue remodeling factors, increased anti-apoptotic genes, alterations in cellular metabolism, endoplasmic reticulum stress, accumulation of lysosomes and mitochondria, and changes in nuclear morphology and composition [3]. Despite all of these abnormalities, there is no perfect, universal biomarker for cellular senescence. Thus, a panel of biomarkers is commonly used to help decide whether there is cellular senescence [3, 4•] (Table 1).

**Table 1:** Biomarkers of cellular senescence

• Grown arrest	
• DNA damage response—γH2AX, pATM, ATR, p53BP, p53, p21	
• Activation of INK4/ARF locus—p16^INK4a^	
• Senescence-associated secretory phenotype (SASP)—IL1α, IL-6, IL-8, TNFα (and many more)	
• Senescence-associated β-galactosidase (SABG)	
• Senescence-associated heterochromatin foci (SAHF)	

The growth arrest of normal cells after a certain number of divisions has been termed the “Hayflick limit.” In Hayflick’s experiments, cellular senescence was induced by replication, and subsequent studies have shown this is dependent on telomere shortening. Expression of telomerase, which lengthens telomeres, allows cells to continue dividing past their normal Hayflick limit. Cellular senescence is now recognized to occur with a number of other stimuli including expression of oncogenes, genotoxic agents such as radiation or chemotherapy, and chronic cellular stress from a number of factors including hyperglycemia or pro-inflammatory cytokines [3, 4•]. On the other hand, since telomere shortening can also be accelerated by chronic stress, e.g., chronic inflammation, determining the primary mechanism inducing cellular aging in any given clinical situation can be a significant challenge [5].

A common theme in most, but not all types of cellular senescence is DNA damage, especially DNA damage that is persistent [6, 7••]. Persistent DNA damage induces growth arrest, reactive oxygen species (ROS), secretion of pro-inflammatory cytokines, and production of IFNβ [7••]. The cGAS-STING pathway senses cytoplasmic DNA and is a critical link between DNA damage and cellular senescence [8, 9•, 10]. Micronuclei and other cytoplasmic DNA activates cGAS producing GMP–AMP that binds and activates STING. Downstream signals from STING include NFκB and IRF-3 which activate IFNβ and cytokine secretion. Cytoplasmic DNA can also be sensed indirectly via Mitochondrial antiviral signaling (MAVS) protein, a key to sensing cytoplasmic viral RNAs and another potent activator of IFNβ and pro-inflammatory cytokines. Cytoplasmic DNA can be transcribed into RNA by RNA polymerase III. RIG-1 can sense this newly transcribed RNA, activating MAVS [11]. Genotoxic stress can also induce small non-coding RNAs that translocate to the cytoplasm, bind RIG-1, and activate MAVS and IFNβ [12]. In addition, ROS, which are frequently associated with cellular senescence and other forms of cellular stress, can also activate MAVS [13••]. Since IFNβ can induce mitochondrial production of ROS and DNA damage, there can be a ROS-MAVS-IFNβ feedback loop in addition to an ROS-DNA-cGAS/STING or RIG/1MAVS-IFNβ [7••, 14•, 15, 16••].

**Fig. 1:**
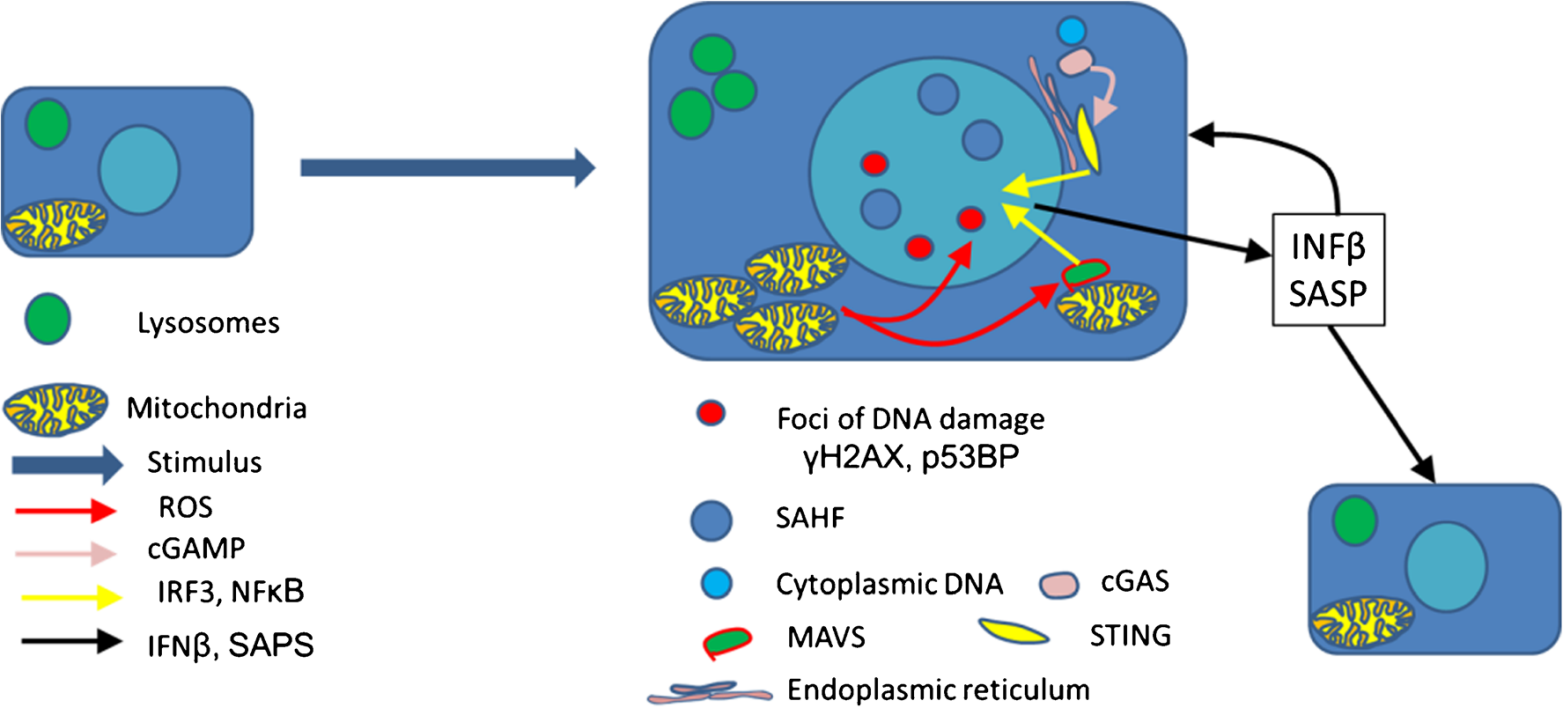
Cellular senescence

Telomeres also mediate damage to DNA. With telomere shortening, telomeric DNA becomes uncapped and more susceptible to damage. However, even without shortening, telomeres are more likely to have persistent DNA damage because the repair process for double-strand break repair is less active in telomeres [17, 18]. Thus, cellular senescence induced by DNA damage may be dependent on telomeres but independent of telomere shortening [19•, 20]. Therefore, while telomere shortening can induce cellular senescence, it is not synonymous with cellular senescence (Fig. 1).

Although senescent cells do not divide, they are metabolically very active. Possibly the most important characteristic of senescent cells is their production of paracrine factors impacting surrounding cells. These factors include a variety of pro-inflammatory cytokines that can recruit inflammatory cells and activate surrounding non-senescent cells, growth factors that can promote tissue repair or growth of neoplastic cells, ROS that can cause DNA damage in surrounding cells, and exosomes that can transfer miRNA and other intracellular macromolecules. These factors can promote senescence of adjacent normal cells. Thus, a small number of senescent cells can have a major impact on function and survival.

Senescent cells permanently withdraw from the cell cycle and they cannot be induced to proliferate. In contrast, cell cycle arrest in quiescent cells is reversible and they can be induced to proliferate [21]. Cellular senescence can be viewed as a multi-step process: (1) cessation of cell growth, (2) metabolic changes regulated by AMPK, (3) activation of mTOR which promotes proliferation of mitochondria, generation of ROS, and secretion of several of the cytokines involved with senescence associated secretory phenotype (SASP), and finally, (4) permanent cell cycle arrest [22•, 23••, 24•]. Therefore, near-senescent cells may have several of the biomarkers of senescent cells but still able to reenter the replication cycle given the right conditions. The transition from temporary to permanent cell cycle arrest has been termed geroconversion [21]. In cells with growth arrest activation of the mTOR pathway appears to play a key role in geroconversion. Inhibitors of mTOR such as rapamycin prevent the transition from temporary to irreversible cell cycle arrest in several models of cellular senescence (overexpression of p21, genotoxic drugs such as etoposide) [25, 26]. Certain conditions of cell culture, e.g., hypoxia or cell confluence inhibition of growth, also prevent geroconversion [27, 28•, 29]. Interestingly, these culture conditions also prevent mTOR activation. Although the mechanisms responsible for permanent, irreversible cell cycle arrest in senescent cells have not been worked out, there is evidence for the involvement of the transcription factor ATRX. With genotoxic stress ATRX localizes to senescence-associated heterochromatin foci (SAHF) [30, 31••, 32]. Reduction of ATRX does not decrease the DNA damage response or the induction of p53 and phosphorylated Rb but it does prevent accumulation of SABG and SAHF positive cells and induction of SASP. Most importantly, silencing ATRX prevents permanent growth arrest. Thus, localization of ATRX to SAHF may provide a marker for permanent cell cycle arrest in cells treated with genotoxic drugs. Whether ATRX plays a role in other types of cellular senescence remains to be seen.

## Why Does Cellular Senescence Matter?

Aging is the major risk factor for mortality and for many of the chronic, non-infectious diseases, e.g., cardiovascular diseases, cancers, Alzheimer’s, Parkinson’s, osteoarthritis, osteoporosis. Cellular senescence also increases with age and appears to play an important role in each of these diseases. Thus, the development of senolytic drugs that specifically target senescent cells as well as the development of drugs that target the senescence-associated secretory phenotype has the potential to revolutionize our treatment of many of these age-associated diseases.

The effects of cellular senescence can extend well beyond the effects on the senescent cells themselves. Senescent cells have a much more general effect locally or systemically through secretion of cytokines, the production of ROA, and the generation of exosomes. Thus, even though senescent cells may be a minor population in a tissue or an organism, their effects can be disproportionate to their number. The potency of senescent cells was seen most clearly in transplantation studies where mice received senescent cells equivalent to 1/10,000 of their own non-senescent cells [33]. Transplanting this small numbers of senescent cells into young mice can cause persistent decrease in physical function. In addition, senescent cell transplantation increased cellular senescence in recipient cells and tissues. Transplanting senescent cells in older mice similarly decreased physical function and also reduced survival. Thus, senescent cells shortened health and life span. Conversely, treating mice with senolytic drugs after transplantation with senescent cells eliminated the transplanted cells and prevented the deleterious effects of the transplant. More impressively, older non-transplanted mice treated with senolytic drugs had improved physical function, increased life-span, and decreased senescent cells. Therapies targeting senescent cells also appear to be beneficial in murine models of age-related diseases, e.g., osteoporosis and cardiovascular disease, or the adverse effects of genotoxic therapies. Elimination of senescent cells using a genetic approach (expression of suicide gene using enhancer for p16^INKa^) or blocking senescence-associated secretory phenotype (SASP) using a JAK inhibitor resulted in higher bone mass and strength and better bone microarchitecture. [34••, 35] Using a genetic elimination of senescent cells in normal-aged mice also improved cardiac histology and resilience to stress and prevented glomerular sclerosis [36, 37].

## Cellular Senescence in Stem Cells

By definition, stem cells cannot be fully senescent. They cannot have permanent cell cycle arrest because they are defined by their ability to divide and repopulate cell populations. Thus, full senescence of stem cells is seen as stem cell exhaustion. Partial senescence of stem cells, i.e., less than full senescence, is generally inferred by quantitative or qualitative abnormalities in their progeny. Telomerase-knockout mice provide one opportunity to observe stem cell senescence [7••]. These animals have distortions in the crypt and villus architecture, and intestinal epithelial cell have increased DNA damage response, elevated p53 and p16^INK4a^, increased SABG expression, decreased proliferation, and increased activation of apoptosis. Telomerase-knockout mice had decreased hematopoietic stems cells as assessed by flow cytometry (lineage-/Sca1+/c-Kit+ LSK stem cells and long-term self-renewing LSK CD150+/CD48- stem cells) and the limited ability of bone marrow to form colonies in vitro. Furthermore, these animals also had skewing towards the myeloid lineage and away from the lymphoid lineage. Interestingly, all the abnormalities in intestinal cells and hematopoietic stem cells in telomerase-knockout mice were absent when the interferon receptor I was also knocked out [7••]. Similar abnormalities in hematopoietic stem cells have been observed in aging animals [38]. Moreover, hematopoietic cells and muscle stem cells prematurely aged by irradiation could be eliminated using a senolytic agent. Treating irradiated animals with a senolytic agent rejuvenating stem cell function [39••].

## Cellular Senescence in Lupus Mesenchymal Stem Cells

The strongest case for cellular senescence in lupus can be made for bone marrow mesenchymal stem cells (BMSC). In part, this is an accident of interest, i.e., this is where investigators have looked. However, BMSC have been a favorite focus in many studies of cellular senescence in part because osteoporosis is such a prevalent disease of aging and in part because BMSC are easy to grow and study in tissue culture. Also, compared to some other organ systems, e.g., the immune system, cellular senescence in BMSC represents a relatively simple system.

During in vitro culture of human, bone marrow mesenchymal stem cells (BMSC) grow very well for 20 population doublings (PD) but develop cellular senescence around 40 PD [40, 41]. Late passage BMSC compared to early passage cells have slower proliferation, markedly increased cell size (area increases from 5 to 50 μm^2^), and increased expression of senescence associated β galactosidase (SABG), p53, and p16^INK4a^. Moreover, late passage BMSC did not repair DNA damage as well as early passage BMSC and were much more sensitive to oxidative stress. Thus, with extended in vitro replication BMSC exhibit the typical finding of cellular senescence. Moreover, BMSC obtained from older donors develop these biomarkers of cellular senescence more rapidly than BMSC from younger donors.

Multiple papers have reported BMSC from lupus patients have many of the same characteristics of BMSC that have undergone replicative senescence. Initial studies found that BMSC from lupus patients compared to matched controls proliferated more slowly, and had a flattened morphology, increased ROS, increased expression of p16^INK4a^, and increased activation of the p53/p21 pathway [42–46].

Downregulation of p16^INK4a^ or p21 mRNA was able to reverse several of these abnormalities [45, 46]. Thus, the biomarkers of senescence in lupus BMSC are at least partial reversible. Since the short-term cultures used in these experiments are a mixture of cells at different stages of aging on their way to cellular senescence, the reversal of biomarkers may have been due to effects on cells early in the course of senescence before changes become irreversible.

Several recent papers have investigated the signaling pathways activated in lupus BMSC and have identified several “druggable targets” including mTOR [47], JAK-STAT [48], and the Wnt/β-catenin pathway [49]. The mTOR inhibitor rapamycin has already been shown to be beneficial for disease activity in murine models of lupus [47]. Rapamycin administration in the MRL/lpr murine model of lupus decreased proteinuria and the number of crescentic glomeruli. Moreover, rapamycin also reversed the cellular senescence of BMSC from lupus mice [47]. In vitro rapamycin treatment of human BMSC from lupus patients also reversed many of the abnormalities associated with cellular senescence including activation of the mTOR pathway, SABG expression, and cellular hypertrophy. BMSC from lupus patients have been shown to be defective in their immunomodulatory activity when transferred to lupus-prone mice [50]. Treating human lupus BMSC with rapamycin reversed this defect and enhanced immunomodulatory effects when transplanted into the MRL/lpr mice [47]. There is enhanced activation of the JAK-STAT pathway in BMSC from lupus patients. Treating lupus BMSC with a JAK2 inhibitor for 48 h. blocked phosphorylation of STAT3, increased proliferation, markedly decreased SABG positive cells. Nuclear levels of β-catenin were found to be markedly elevated in SLE BMSC [49]. Wnt/β-catenin pathway inhibition in SLE BMSC using Dickkopf WNT Signaling Pathway Inhibitor 1 (DKK1) or β-catenin siRNA reversed many of the biomarkers of cellular senescence. For example, Wnt/β-catenin pathway inhibition increased proliferation, decreased the number of SABG positive cells, and decreased expression of p53 and p21.

Our own studies have found that lupus BMSC have many of the hallmarks of cellular senescence including a reduced proliferation rate, increased DNA damage and repair, increased production of reactive oxygen species, increased expression of p53 and p16, and increased secretion of pro-inflammatory cytokines. Notably, SLE BM-MSCs had a 5-fold increase in interferon-β (IFNβ) levels and increased IFNβ-induced messenger RNAs (mRNAs). In addition, MAVS expression was elevated, and the level of MAVS was highly correlated with IFNβ levels (*r* > 0.9, *P* < 0.01). Since MAVS is known to be a potent inducer of IFNβ, we hypothesized that there is a positive feedback loop between MAVS and IFNβ. We also found that silencing MAVS markedly decreased IFNβ, p53, and p16 protein levels and expression of mRNA for proinflammatory cytokines. Increased levels of IFNβ are seen in human diploid fibroblasts from patients with Werner or Hutchinson-Gilford progeria syndromes compared to normal controls [7••]. Increased levels of IFNβ have also been seen in late passage compared to early passage human fibroblast. Treatment of late passage normal fibroblasts or fibroblast from patients with progeria syndrome with anti-IFNβ increased cell proliferation, decreased cells positive for SABG, and decreased p16, p21, and p53 protein expression.

In summary, multiple investigators have found human BMSC from lupus patients have the hallmarks of cellular senescence and have demonstrated potential therapies that reverse these biomarkers in vitro. Thus, there is a strong case for accelerated cellular senescence in lupus MSC and a strong case for the potential of interventions. However, the clinical significance of BMSC cellular senescence in lupus is still unclear. Moreover, whether using any of these interventions will reverse the abnormalities in lupus BMSC in vivo remains an open question.

## Cellular Senescence in Organ Systems with Lupus

DNA damage, a key inducer of cellular senescence, is associated with SLE in multiple studies [51–53, 54••]. Metabolic abnormalities and increased production of mitochondrial reactive oxygen species are part of the problems but defects in DNA repair have also been seen [13••, 52, 55, 56]. Thus, cellular stress with increased reactive oxygen species and DNA damage is a major feature of SLE and may contribute to accelerated cellular senescence.

Markers of cellular senescence have been reported in many types of renal disease including several types of glomerular nephritis [57•, 58•, 59, 60•, 61]. Moreover, knocking out one of the primary pathways of cellular senescence prevents interstitial fibrosis and tubular atrophy with renal injury, in transplanted kidneys, or in kidneys with premature aging [62, 63]. Interestingly, evidence of senescence in the kidney can occur without evidence of telomere shortening [64]. Very recently, data shows biomarkers of cellular senescence in murine and human lupus including upregulation of SABG and p16^ink4a^ [65••, 66]. These studies have shown a potential role for wnt9a in renal cellular senescence and fibrosis [65••]. As discussed above, wnt signaling has also been shown to play a role in cellular senescence of lupus BMSC [49]. Thus, there is a reasonable chance that cellular senescence will play an important role in lupus nephritis, which is one of the most intractable manifestations of lupus.

Cardiovascular disease, the leading cause of death in developed countries, is closely associated with aging, and there is a strong case for cellular senescence mediating initiation and progression of cardiovascular disease [37]. Inducers of cellular senescence such as telomere shortening and oxidative stress are associated with age and cells expressing biomarkers of aging accumulate in the heart and blood vessels. Moreover, in animal models, genetic or pharmacologic elimination of senescence cells block the effect of aging on cardiovascular function and the development of cardiovascular disease. Lupus is well known to have a striking excess of atherosclerotic disease [67, 68]. A recent nationwide cohort study in Korea found that lupus is associated with markedly increased hazard ratio for myocardial infarction (2.74), stroke (3.31), heart failure (4.60) and cardiac death (3.98) [69]. However, to the best of our knowledge, excess cardiovascular disease in lupus has not yet been linked to accelerated cellular senescence.

A new twist to the story of cellular senescence involves post-mitotic cells such as neurons. Neurons in old mice show several hallmarks of cellular senescence such as severe DNA damage, production of ROA, secretion of pro-inflammatory cytokines, IL-6 production, and senescence-associated β-galactosidase activity [70]. Moreover, these changes were dependent on p21 which is also a necessary link between the DNA damage response and senescence-like phenotype in proliferative cells such as fibroblasts. Thus, it will be interesting to see if drugs targeting cellular senescence, e.g., drugs inhibiting SASP, are also beneficial in models of age-associated CNS disease. On the other hand, this observation suggests senolytic drugs, drugs that induce apoptosis in senescing cells, might have unintended adverse effects on neurons. Initial studies of senolytic drugs in a murine model of Alzheimer’s look promising but much more work is needed [71••, 72]. CNS disease in lupus is extremely heterogeneous and probably has several pathogenic mechanisms including autoantibodies and type I interferon [73–75]. However, to the best of our knowledge, cellular senescence in the CNS has not been evaluated in lupus.

The changes in the innate and adaptive immune system seen in lupus are complex. There is a recent review that does a superb job discussing the similarities and differences between the immune changes seen with lupus and the immune changes seen with aging [76••]. Suffice to say that although there are many similarities at the molecular levels, there are major differences in function, i.e., the immune changes in lupus are very distinct from immunosenescence.

## Summary

SLE is a chronic inflammatory disease that affects all major organ systems. Inflammation has long been proposed as a cause for accelerated aging. At this point, there is a moderate amount of evidence indicating cellular senescence may play an important role in lupus. However, not all inflammation is the same and not all cells with biomarkers of senescence have reached the stage where changes are irreversible. Therefore, interventions targeting interferons and pro-inflammatory cytokines or signaling pathways may be able to reverse many of the abnormalities suggesting cellular senescence in patients with lupus. Alternatively, it may be possible using senolytic drugs to selectively eliminate senescence cells the rejuvenate tissues and organs adversely affected by lupus.

## References

[1] Koopman JJ (2011). Senescence rates in patients with end-stage renal disease: a critical appraisal of the Gompertz model. Aging Cell.

[2] Lopez-Otin C (2013). The hallmarks of aging. Cell.

[3] Hernandez-Segura A, Nehme J, Demaria M (2018). Hallmarks of cellular senescence. Trends Cell Biol.

[4] Herranz N, Gil J (2018). Mechanisms and functions of cellular senescence. J Clin Investig.

[5] Jose SS, Bendickova K, Kepak T, Krenova Z, Fric J (2017). Chronic inflammation in immune aging: role of pattern recognition receptor crosstalk with the telomere complex?. Front Immunol.

[6] Bielak-Zmijewska A, Mosieniak G, Sikora E (2018). Is DNA damage indispensable for stress-induced senescence?. Mech Ageing Dev.

[7] Yu Q (2015). DNA-damage-induced type I interferon promotes senescence and inhibits stem cell function. Cell Rep.

[8] Yang H, Wang H, Ren J, Chen Q, Chen ZJ (2017). cGAS is essential for cellular senescence. Proc Natl Acad Sci U S A.

[9] Li T, Chen ZJ (2018). The cGAS-cGAMP-STING pathway connects DNA damage to inflammation, senescence, and cancer. J Exp Med.

[10] Cai X, Chiu YH, Chen ZJ (2014). The cGAS-cGAMP-STING pathway of cytosolic DNA sensing and signaling. Mol Cell.

[11] Chiu YH, Macmillan JB, Chen ZJ (2009). RNA polymerase III detects cytosolic DNA and induces type I interferons through the RIG-I pathway. Cell.

[12] Ranoa DR, Parekh AD, Pitroda SP, Huang X, Darga T, Wong AC, Huang L, Andrade J, Staley JP, Satoh T, Akira S, Weichselbaum RR, Khodarev NN (2016). Cancer therapies activate RIG-I-like receptor pathway through endogenous non-coding RNAs. Oncotarget.

[13] Buskiewicz IA (2016). Reactive oxygen species induce virus-independent MAVS oligomerization in systemic lupus erythematosus. Sci Signal.

[14] Meyer A (2017). IFN-beta-induced reactive oxygen species and mitochondrial damage contribute to muscle impairment and inflammation maintenance in dermatomyositis. Acta Neuropathol.

[15] Agod Z, Fekete T, Budai MM, Varga A, Szabo A, Moon H, Boldogh I, Biro T, Lanyi A, Bacsi A, Pazmandi K (2017). Regulation of type I interferon responses by mitochondria-derived reactive oxygen species in plasmacytoid dendritic cells. Redox Biol.

[16] Moiseeva O (2006). DNA damage signaling and p53-dependent senescence after prolonged beta-interferon stimulation. Mol Biol Cell.

[17] Fumagalli M, Rossiello F, Clerici M, Barozzi S, Cittaro D, Kaplunov JM, Bucci G, Dobreva M, Matti V, Beausejour CM, Herbig U, Longhese MP, d’Adda di Fagagna F (2012). Telomeric DNA damage is irreparable and causes persistent DNA-damage-response activation. Nat Cell Biol.

[18] Hewitt G, Jurk D, Marques FDM, Correia-Melo C, Hardy T, Gackowska A, Anderson R, Taschuk M, Mann J, Passos JF (2012). Telomeres are favoured targets of a persistent DNA damage response in ageing and stress-induced senescence. Nat Commun.

[19] Victorelli S, Passos JF (2017). Telomeres and cell senescence-size matters not. EBioMedicine.

[20] Birch J, Barnes PJ, Passos JF (2018). Mitochondria, telomeres and cell senescence: implications for lung ageing and disease. Pharmacol Ther.

[21] Blagosklonny MV (2014). Geroconversion: irreversible step to cellular senescence. Cell Cycle.

[22] Rodier F, Campisi J (2011). Four faces of cellular senescence. J Cell Biol.

[23] Laberge RM (2015). MTOR regulates the pro-tumorigenic senescence-associated secretory phenotype by promoting IL1A translation. Nat Cell Biol.

[24] Wiley CD, Campisi J (2016). From ancient pathways to aging cells-connecting metabolism and cellular senescence. Cell Metab.

[25] Sousa-Victor P, Garcia-Prat L, Munoz-Canoves P (2015). Dual mTORC1/C2 inhibitors: gerosuppressors with potential anti-aging effect. Oncotarget.

[26] Demidenko ZN, Zubova SG, Bukreeva EI, Pospelov VA, Pospelova TV, Blagosklonny MV (2009). Rapamycin decelerates cellular senescence. Cell Cycle.

[27] Leontieva OV, Demidenko ZN, Blagosklonny MV (2014). Contact inhibition and high cell density deactivate the mammalian target of rapamycin pathway, thus suppressing the senescence program. Proc Natl Acad Sci U S A.

[28] Leontieva OV (2012). Hypoxia suppresses conversion from proliferative arrest to cellular senescence. Proc Natl Acad Sci U S A.

[29] Leontieva OV, Blagosklonny MV (2012). Hypoxia and gerosuppression: the mTOR saga continues. Cell Cycle.

[30] Kovatcheva M, Klein ME, Tap WD, Koff A (2018). Mechanistic understanding of the role of ATRX in senescence provides new insight for combinatorial therapies with CDK4 inhibitors. Mol Cell Oncol.

[31] Kovatcheva M (2017). ATRX is a regulator of therapy induced senescence in human cells. Nat Commun.

[32] Kovatcheva M, Liu DD, Dickson MA, Klein ME, O'Connor R, Wilder FO, Socci ND, Tap WD, Schwartz GK, Singer S, Crago AM, Koff A (2015). MDM2 turnover and expression of ATRX determine the choice between quiescence and senescence in response to CDK4 inhibition. Oncotarget.

[33] Xu M, Pirtskhalava T, Farr JN, Weigand BM, Palmer AK, Weivoda MM, Inman CL, Ogrodnik MB, Hachfeld CM, Fraser DG, Onken JL, Johnson KO, Verzosa GC, Langhi LGP, Weigl M, Giorgadze N, LeBrasseur NK, Miller JD, Jurk D, Singh RJ, Allison DB, Ejima K, Hubbard GB, Ikeno Y, Cubro H, Garovic VD, Hou X, Weroha SJ, Robbins PD, Niedernhofer LJ, Khosla S, Tchkonia T, Kirkland JL (2018). Senolytics improve physical function and increase lifespan in old age. Nat Med.

[34] Farr JN (2017). Targeting cellular senescence prevents age-related bone loss in mice (vol 23, pg 1072, 2017). Nat Med.

[35] Khosla S, Farr JN, Kirkland JL (2018). Inhibiting cellular senescence: a new therapeutic paradigm for age-related osteoporosis. J Clin Endocrinol Metab.

[36] Baker DJ, Childs BG, Durik M, Wijers ME, Sieben CJ, Zhong J, A. Saltness R, Jeganathan KB, Verzosa GC, Pezeshki A, Khazaie K, Miller JD, van Deursen JM (2016). Naturally occurring p16(Ink4a)-positive cells shorten healthy lifespan. Nature.

[37] Childs BG, Li H, van Deursen JM (2018). Senescent cells: a therapeutic target for cardiovascular disease. J Clin Invest.

[38] Denkinger MD, Leins H, Schirmbeck R, Florian MC, Geiger H (2015). HSC aging and senescent immune remodeling. Trends Immunol.

[39] Chang J (2016). Clearance of senescent cells by ABT263 rejuvenates aged hematopoietic stem cells in mice. Nat Med.

[40] Yu J, Shi J, Zhang Y, Zhang Y, Huang Y, Chen Z, Yang J (2018). The replicative senescent mesenchymal stem/stromal cells defect in DNA damage response and anti-oxidative capacity. Int J Med Sci.

[41] Ganguly P, el-Jawhari JJ, Giannoudis PV, Burska AN, Ponchel F, Jones EA (2017). Age-related changes in bone marrow mesenchymal stromal cells: a potential impact on osteoporosis and osteoarthritis development. Cell Transplant.

[42] Sun LY, Zhang HY, Feng XB, Hou YY, Lu LW, Fan LM (2007). Abnormality of bone marrow-derived mesenchymal stem cells in patients with systemic lupus erythematosus. Lupus.

[43] Nie Y, Lau C, Lie A, Chan G, Mok M (2010). Defective phenotype of mesenchymal stem cells in patients with systemic lupus erythematosus. Lupus.

[44] Li X, Liu L, Meng D, Wang D, Zhang J, Shi D, Liu H, Xu H, Lu L, Sun L (2012). Enhanced apoptosis and senescence of bone-marrow-derived mesenchymal stem cells in patients with systemic lupus erythematosus. Stem Cells Dev.

[45] Gu Z (2013). p53/p21 pathway involved in mediating cellular senescence of bone marrow-derived mesenchymal stem cells from systemic lupus erythematosus patients. Clin Dev Immunol.

[46] Gu Z, Cao X, Jiang J, Li L, da Z, Liu H, Cheng C (2012). Upregulation of p16INK4A promotes cellular senescence of bone marrow-derived mesenchymal stem cells from systemic lupus erythematosus patients. Cell Signal.

[47] Gu Z, Tan W, Ji J, Feng G, Meng Y, da Z, Guo G, Xia Y, Zhu X, Shi G, Cheng C (2016). Rapamycin reverses the senescent phenotype and improves immunoregulation of mesenchymal stem cells from MRL/lpr mice and systemic lupus erythematosus patients through inhibition of the mTOR signaling pathway. Aging (Albany NY).

[48] Ji J, Wu Y, Meng Y, Zhang L, Feng G, Xia Y, Xue W, Zhao S, Gu Z, Shao X (2017). JAK-STAT signaling mediates the senescence of bone marrow-mesenchymal stem cells from systemic lupus erythematosus patients. Acta Biochim Biophys Sin Shanghai.

[49] Gu Z, Tan W, Feng G, Meng Y, Shen B, Liu H, Cheng C (2014). Wnt/beta-catenin signaling mediates the senescence of bone marrow-mesenchymal stem cells from systemic lupus erythematosus patients through the p53/p21 pathway. Mol Cell Biochem.

[50] Collins E, Gu F, Qi M, Molano I, Ruiz P, Sun L, Gilkeson GS (2014). Differential efficacy of human mesenchymal stem cells based on source of origin. J Immunol.

[51] Souliotis VL, Sfikakis PP (2015). Increased DNA double-strand breaks and enhanced apoptosis in patients with lupus nephritis. Lupus.

[52] Souliotis VL, Vougas K, Gorgoulis VG, Sfikakis PP (2016). Defective DNA repair and chromatin organization in patients with quiescent systemic lupus erythematosus. Arthritis Res Ther.

[53] Davies RC, Pettijohn K, Fike F, Wang J, Nahas SA, Tunuguntla R, Hu H, Gatti RA, McCurdy D (2012). Defective DNA double-strand break repair in pediatric systemic lupus erythematosus. Arthritis Rheum.

[54] Gao L (2017). Bone marrow-derived mesenchymal stem cells from patients with systemic lupus erythematosus have a senescence-associated secretory phenotype mediated by a mitochondrial antiviral signaling protein-interferon-beta feedback loop. Arthritis Rheumatol.

[55] Perl A, Banki K (2000). Genetic and metabolic control of the mitochondrial transmembrane potential and reactive oxygen intermediate production in HIV disease. Antioxid Redox Signal.

[56] Banki K (1999). Elevation of mitochondrial transmembrane potential and reactive oxygen intermediate levels are early events and occur independently from activation of caspases in Fas signaling. J Immunol.

[57] Valentijn FA (2018). Cellular senescence in the aging and diseased kidney. J Cell Commun Signal.

[58] Sis B (2007). Accelerated expression of senescence associated cell cycle inhibitor p16INK4A in kidneys with glomerular disease. Kidney Int.

[59] Melk A, Schmidt BMW, Vongwiwatana A, Rayner DC, Halloran PF (2005). Increased expression of senescence-associated cell cycle inhibitor p16INK4a in deteriorating renal transplants and diseased native kidney. Am J Transplant.

[60] Sturmlechner I (2017). Cellular senescence in renal ageing and disease. Nat Rev Nephrol.

[61] Verzola D, Gandolfo MT, Gaetani G, Ferraris A, Mangerini R, Ferrario F, Villaggio B, Gianiorio F, Tosetti F, Weiss U, Traverso P, Mji M, Deferrari G, Garibotto G (2008). Accelerated senescence in the kidneys of patients with type 2 diabetic nephropathy. Am J Physiol Ren Physiol.

[62] Braun H, Schmidt BMW, Raiss M, Baisantry A, Mircea-Constantin D, Wang S, Gross ML, Serrano M, Schmitt R, Melk A (2012). Cellular senescence limits regenerative capacity and allograft survival. J Am Soc Nephrol.

[63] Jin J, Tao J, Gu X, Yu Z, Wang R, Zuo G, Li Q, Lv X, Miao D (2017). P16 (INK4a) deletion ameliorated renal Tubulointerstitial injury in a stress-induced premature senescence model of Bmi-1 deficiency. Sci Rep.

[64] Melk A, Kittikowit W, Sandhu I, Halloran KM, Grimm P, Schmidt BMW, Halloran PF (2003). Cell senescence in rat kidneys in vivo increases with growth and age despite lack of telomere shortening. Kidney Int.

[65] Luo C (2018). Wnt9a promotes renal fibrosis by accelerating cellular senescence in tubular epithelial cells. J Am Soc Nephrol.

[66] Yang C, Xue J, An N, Huang XJ, Wu ZH, Ye L, Li ZH, Wang SJ, Pan QJ, Liang D, Liu HF (2018). Accelerated glomerular cell senescence in experimental lupus nephritis. Med Sci Monit.

[67] Roman MJ, Shanker BA, Davis A, Lockshin MD, Sammaritano L, Simantov R, Crow MK, Schwartz JE, Paget SA, Devereux RB, Salmon JE (2003). Prevalence and correlates of accelerated atherosclerosis in systemic lupus erythematosus. N Engl J Med.

[68] Asanuma Y, Oeser A, Shintani AK, Turner E, Olsen N, Fazio S, Linton MRF, Raggi P, Stein CM (2003). Premature coronary-artery atherosclerosis in systemic lupus erythematosus. N Engl J Med.

[69] Lim S Y, Bae E H, Han K-D, Jung J-H, Choi H S, Kim HY, Kim C S, Ma S K, Kim S W (2018). Systemic lupus erythematosus is a risk factor for cardiovascular disease: a nationwide, population-based study in Korea. Lupus.

[70] Jurk D, Wang C, Miwa S, Maddick M, Korolchuk V, Tsolou A, Gonos ES, Thrasivoulou C, Jill Saffrey M, Cameron K, von Zglinicki T (2012). Postmitotic neurons develop a p21-dependent senescence-like phenotype driven by a DNA damage response. Aging Cell.

[71] Bussian Tyler J., Aziz Asef, Meyer Charlton F., Swenson Barbara L., van Deursen Jan M., Baker Darren J. (2018). Clearance of senescent glial cells prevents tau-dependent pathology and cognitive decline. Nature.

[72] Musi N, Valentine JM, Sickora KR, Baeuerle E, Thompson CS, Shen Q, Orr ME (2018). Tau protein aggregation is associated with cellular senescence in the brain. Aging Cell.

[73] McGlasson S, Wiseman S, Wardlaw J, Dhaun N, Hunt DPJ (2018). Neurological disease in lupus: toward a personalized medicine approach. Front Immunol.

[74] Nestor J, Arinuma Y, Huerta TS, Kowal C, Nasiri E, Kello N, Fujieda Y, Bialas A, Hammond T, Sriram U, Stevens B, Huerta PT, Volpe BT, Diamond B (2018). Lupus antibodies induce behavioral changes mediated by microglia and blocked by ACE inhibitors. J Exp Med.

[75] Bialas AR, Presumey J, Das A, van der Poel C, Lapchak PH, Mesin L, Victora G, Tsokos GC, Mawrin C, Herbst R, Carroll MC (2017). Microglia-dependent synapse loss in type I interferon-mediated lupus. Nature.

[76] van den Hoogen LL (2015). Aging and systemic lupus erythematosus-immunosenescence and beyond. Curr Aging Sci.

